# Therapeutic Validation of GEF-H1 Using a De Novo Designed Inhibitor in Models of Retinal Disease

**DOI:** 10.3390/cells11111733

**Published:** 2022-05-24

**Authors:** Clare Mills, Sandra A. Hemkemeyer, Zerin Alimajstorovic, Chantelle Bowers, Malihe Eskandarpour, John Greenwood, Virginia Calder, A. W. Edith Chan, Paul J. Gane, David L. Selwood, Karl Matter, Maria S. Balda

**Affiliations:** 1 UCL Institute of Ophthalmology, University College London, 11-43 Bath Street, London EC1V 9EL, UK; claremills@hotmail.co.uk (C.M.); s.hemkemeyer@uke.de (S.A.H.); zerin.alimajstorovic@googlemail.com (Z.A.); chantelle.bowers.13@ucl.ac.uk (C.B.); m.eskandarpour@ucl.ac.uk (M.E.); j.greenwood@ucl.ac.uk (J.G.); v.calder@ucl.ac.uk (V.C.); 2 The Wolfson Institute for Biomedical Research, University College London, Gower Street, London WC1E 6BT, UK; edith.chan@ucl.ac.uk (A.W.E.C.); p.gane@ucl.ac.uk (P.J.G.); d.selwood@ucl.ac.uk (D.L.S.)

**Keywords:** RhoA, tight junctions, inflammation, retinal pigment epithelium, endothelium

## Abstract

Inflammation and fibrosis are important components of diseases that contribute to the malfunction of epithelia and endothelia. The Rho guanine nucleotide exchange factor (GEF) GEF-H1/ARHGEF-2 is induced in disease and stimulates inflammatory and fibrotic processes, cell migration, and metastasis. Here, we have generated peptide inhibitors to block the function of GEF-H1. Inhibitors were designed using a structural in silico approach or by isolating an inhibitory sequence from the autoregulatory C-terminal domain. Candidate inhibitors were tested for their ability to block RhoA/GEF-H1 binding in vitro, and their potency and specificity in cell-based assays. Successful inhibitors were then evaluated in models of TGFβ-induced fibrosis, LPS-stimulated endothelial cell-cell junction disruption, and cell migration. Finally, the most potent inhibitor was successfully tested in an experimental retinal disease mouse model, in which it inhibited blood vessel leakage and ameliorated retinal inflammation when treatment was initiated after disease diagnosis. Thus, an antagonist that blocks GEF-H1 signaling effectively inhibits disease features in in vitro and in vivo disease models, demonstrating that GEF-H1 is an effective therapeutic target and establishing a new therapeutic approach.

## 1. Introduction

Epithelia and endothelia are fundamental for the structure and function of organs and complex tissues. They form cellular barriers that shield our bodies and separate body compartments. An example of striking importance is the retinal pigment epithelium (RPE), which forms the posterior blood-retinal barrier and is essential for retinal function and homeostasis [1]. Pathological responses involving the malfunction of epithelia and endothelia are important components of many clinically relevant diseases and often lead to the loss of organ function. In the eye, diseases such as infections, age-related macular degeneration, diabetes, and glaucoma, as well as mechanical traumas lead to reduced or loss of vision due to malfunctioning epithelial and endothelial cells [1,2,3,4,5]. Although such diverse disease conditions are triggered by different stimuli and involve various molecular mechanisms, they share certain fundamental subcellular signaling mechanisms. One such signaling mechanism centers on GEF-H1/ARHGEF2, a guanine nucleotide exchange factor for RhoA (GEF). GEF-H1 regulates crosstalk between microtubules and the actin cytoskeleton, a process thought to be important during cell contractility and migration, cell shape changes, and intercellular junction remodeling, as well as cell proliferation and mitosis [6,7,8,9,10,11,12,13,14,15]. GEF-H1 also stimulates RhoA activation to promote fibrotic and inflammatory responses [16,17,18,19,20,21,22].

RhoA is an important regulatory switch of physiological and pathological pathways that guide processes such as cell migration, gene expression, proliferation, inflammation, and fibrosis. RhoA activation has been characterized in different types of disease and several inhibitors for Rho kinases (ROCKs), key targets of active RhoA, have hence been developed. ROCK inhibitors are in clinical use for pulmonary hypertension, amyotrophic lateral sclerosis, diabetic macular edema, and glaucoma [23,24,25,26,27]. However, ROCKs are involved in multiple homeostatic biological processes; therefore, their inhibition often fails. A possible problem of ROCK inhibition is that ROCKs function in distinct RhoA signaling mechanisms that can have counteracting functions. For example, ROCKs act in cell migration, stress fiber formation, and junction disruption in epithelia, but are also involved in regulating junction formation and resistance to mechanical stress [28,29]. Moreover, ROCKs just represent one single RhoA effector mechanism and other RhoA effectors contribute to the regulation of cytoskeletal remodeling and gene expression. Thus, given the importance of the RhoA activation in different types of physiological and pathological signaling mechanisms, the next generation of RhoA signaling inhibitors has to be more specific for particular processes and target RhoA in a manner that impacts multiple downstream pathways relevant for such processes.

RhoA signaling functions are regulated by different GEFs. More than 80 Rho GEFs have been identified, 70 have DH and PH domains that form a catalytic module, and 10 have the Dedicator of Cytokinesis (DOCK) domain that catalyzes guanine nucleotide exchange [30]. GEF-H1 is one of the DH domain GEFs. RhoA and many of its effectors drive multiple processes in different pathways; hence, it is difficult to block them in a process-specific manner. In contrast, RhoA GEFs are more process-specific [29]. GEF-H1 is such an activator that stimulates RhoA to regulate epithelial and endothelial barrier functions as well as cell migration [10,31,32,33,34]. The process-specificity is well illustrated by GEF-H1 and a counteracting RhoA GEF, p114RhoGEF; both activate ROCK to promote actomyosin contractility. However, p114RhoGEF regulates junction formation and stability, whereas GEF-H1 is activated to promote junction dissociation, epithelial-mesenchymal transition (EMT), and cell migration [29]. Hence, targeting specific GEFs is a more desirable strategy to generate Rho signaling inhibitors that are more process-specific and will impact on all RhoA downstream pathways activated in such a process. To our knowledge, only a few GEF inhibitors have thus far been developed—none for GEF-H1—and there are no GEF inhibitors in clinical trials [35].

Inflammation and fibrosis are common hallmarks of diseases that lead to degeneration and malfunction of epithelia and endothelia and induce drastic changes in the actin cytoskeleton and cell-cell adhesion. GEF-H1 is a crucial component of inflammatory responses triggered by cytokines and infections. This includes TGFβ-induced dedifferentiation, LPS (lipopolysaccharide), as well as oncogenic Ras and p53 signaling leading to tumor cell invasion [17,36,37,38,39]. Many adult tissues express little if any GEF-H1 under normal conditions but upregulate its expression in response to pathological stimuli. This is exemplified by the RPE, in which GEF-H1 is upregulated in patients in response to mechanical traumas and uveitis [17]. Upregulation occurs in response to TGFβ1 signaling and mediates α-smooth muscle actin (αSMA) induction, linking GEF-H1 to fibrotic responses. Inactivation of GEF-H1 inhibits such pathological processes without affecting epithelial integrity and barrier formation [17]. Hence, GEF-H1 is an ideal candidate for a therapeutic target.

Here, we focus on the development of specific inhibitors for GEF-H1. We report the identification of peptide inhibitors that block signaling by GEF-H1 but not closely related RhoA GEFs. The most effective inhibitor, TAT-P5, was found to block GEF-H1-driven epithelial and endothelial cytoskeletal remodeling and malfunction in in vitro disease models for TGFβ1-stimulated fibrosis in primary RPE and LPS-activated processes in primary endothelial cells, as well as cancer cell migration. Finally, TAT-P5 effectively inhibited disease progression in a model of retinal disease in mouse (experimental autoimmune uveitis, EAU) starting inhibitor treatment after disease onset. Thus, GEF-H1 can be successfully inhibited for therapeutic applications, and TAT-P5 represents an important step towards its therapeutic inhibition to treat common diseases affecting epithelia and endothelia.

## 2. Materials and Methods

### 2.1. GEF-H1 Inhibitors

**Figure 1:**
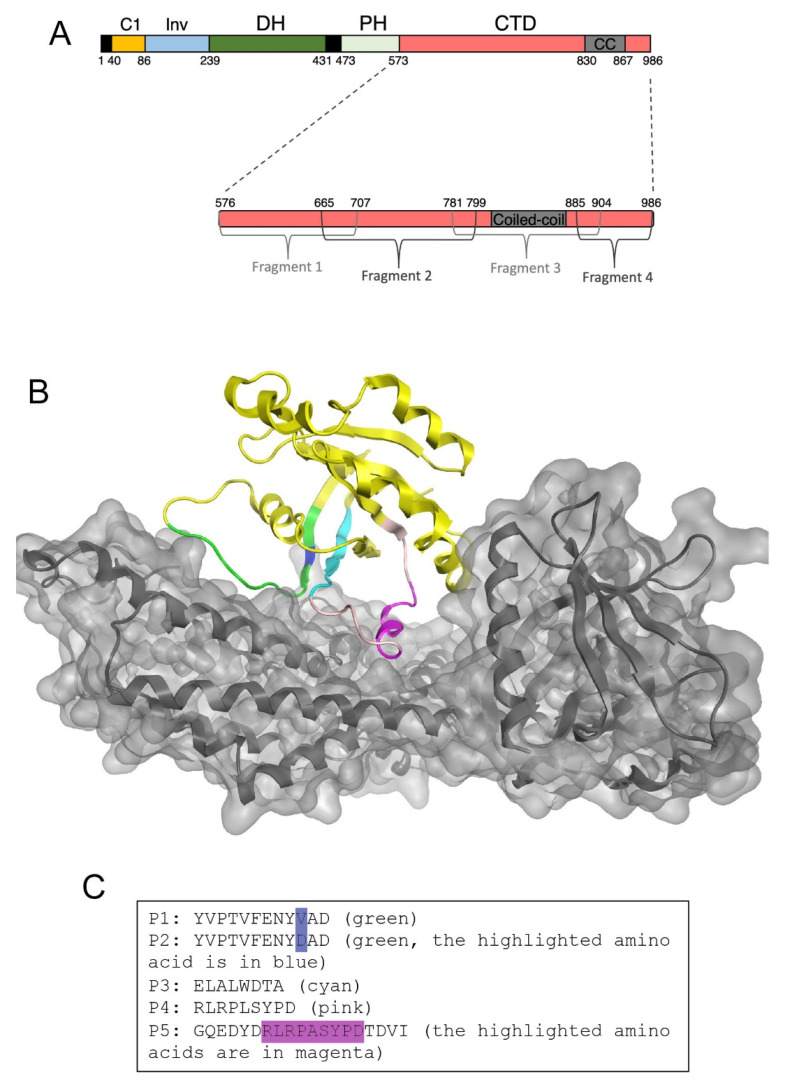
Design of GEF-H1 inhibitors. (**A**) Domain structure of GEF-H1. Indicated are the C1 domain, an intervening (Inv) domain linking the C1 to the Dbl homology domain (DH), the Pleckstrin homology domain (PH), and the C-terminal auto-inhibitory domain (CTD). Removal of the C1 domain and CTD domain leads to a constitutive active mutant (GEF-H1-CA; consisting of Inv, DH, and PH domain) as the C1 domain mediates sequestration of GEF-H1 to microtubules and the CTD contains an auto-inhibitory activity. The inhibitory domain was divided into four fragments that were tested for GEF-H1 inhibition to localize the inhibitory activity in GEF-H1′s CTD. (**B**) Structural modeling of the DH/PH module of GEF-H1 with RhoA. The sequences corresponding to the DH/PH module of GEF-H1 were fitted into the existing crystal structures of RhoA-bound to other Dbl family guanine nucleotide exchange factors (1XCG). The secondary structure of RhoA is represented in yellow ribbons. P1 and P2 peptides are in green; P3 in cyan, and P4 and P5 are in pink/cyan. GEF-H1 is in grey. The resulting interaction surface of GEF-H1 with RhoA was used to select sequences of RhoA likely to be important for binding of RhoA to GEF-H1. (**C**) The sequences of the peptides inhibitors derived from the structural model that were synthesized for experimental validation.

Plasmids encoding the CTD fragments (F1, F2, F3, F4) were generated by PCR using full-length GEF-H1 cDNA as a template. Fragments were cloned into the plasmids pcDNA-TO-VSV for mammalian cell transfection, adding a C-terminal VSV tag for detection and pGEX-4T-3 for recombinant protein expression with an N-terminal GST tag. To generate TAT-F1 and TAT-GST, primers containing the sequences for TAT and the HA tag (Table S1) were annealed and cloned into the pGEX-4T-3 plasmid. The cDNA encoding F1 was then cloned into this plasmid to produce TAT-F1. For recombinant protein production of F1, F2, F3, TAT-GST, and TAT-F1 BL-21pLysS E. Coli were transformed with the respective constructs. Liquid cultures in Luria broth were then grown overnight. Bacterial cultures were induced with 0.5 mM IPTG for 3 h at 30 °C prior to purification of the GST-tagged fusion proteins using standard methods. The purified proteins were dialyzed against PBS containing 10 mM DTT. Endotoxins were removed from purified TAT-GST and TAT-F1 fractions using endotoxin removal spin columns (Pierce) before addition to any mammalian cell cultures. TAT-P5, TAT-Scr (Table S2), and P1-5 (Figure 1C) were generated by chemical synthesis (Generon Ltd., Slough, UK).

### 2.2. RhoA Binding Assay

Purified recombinant GST-RhoA was produced by transforming BL-21pLysS E. Coli with pGEX-4T-3 containing RhoA cDNA. Recombinant protein was then expressed and purified as described above for other GST-tagged proteins. Pulldown beads were prepared by incubating purified GST-RhoA with glutathione beads for 2 h. Beads were then washed with PBS containing 1% Triton X-100 and 1 mM DTT before incubation with PBS containing 5 mM EDTA and 1 mM DTT for 5 min. MDCK cells expressing a constitutively active mutant that lacks the N-terminal C1 domain and the C-terminal domain (ca-GEF-H1-VSV) under the control of a tetracycline inducible promoter were grown to confluence in 9 cm dishes and incubated with tetracycline overnight [13]. The cells were then lysed on ice in PBS containing 1% Triton X-100, 1 mM DTT, and protease inhibitors. Lysates were supplemented with 5 mM EDTA and relevant GEF-H1 inhibitor before addition to the prepared GST-RhoA bound beads. After a one-hour incubation, beads were washed with lysis buffer before boiling in SDS-PAGE sample buffer. Samples were then analyzed by immunoblotting.

### 2.3. Cell Culture and RPE Cell Isolation

HEK293 and MDCK cells were cultured in high glucose Dulbecco’s Modified Eagle Medium (DMEM) containing 10% fetal bovine serum (FBS) and 1% Penicillin/Streptomycin. MDCK cells stably transfected with GEF-H1-VSV, GEF-H1-HA, and p114-VSV under the control of a tetracycline-inducible manner were described previously [10,13,28]. These cell lines were maintained in a medium supplemented with 5 µg/mL Blasticidin (PAA Laboratories) and 400 µg/mL Zeocin (Invitrogen, Waltham, MA, USA). Tetracycline was added at 2 µg/mL to induce construct expression. Cell lines were regularly tested for mycoplasma by DNA staining. Primary HDMECs (PromoCell) were cultured in endothelial cell growth medium MV2 (PromoCell, Heidelberg, Germany) supplemented with C-39225 supplement mix (PromoCell) [40]. Cells were used between passage two and four. Primary porcine RPE cells were isolated from porcine eyes [17]. Upon arrival, eyes were trimmed of excess tissue and washed in Videne antiseptic solution (Ecolab Ltd., St. Paul, MN, USA) diluted 1.4 times in PBS. Eyes were then rinsed in PBS and transferred to a solution of PBS, 33.3% Penicillin/Streptomycin. The lens was removed by cutting with a scalpel at the ora serrata, and the vitreous was discarded. Eye cups were placed into 12-well plates and washed repeatedly with PBS until the neural retina was removed. Eye cups were then washed once with 10× trypsin, refilled with 10× trypsin, and incubated at 37 °C for 30 min. RPE cells were then gently detached by pipetting. Isolated cells were resuspended in 10% FBS DMEM, centrifuged, and resuspended again in 10% FBS DMEM, 1% Penicillin/Streptomycin, 1% Gentamycin, and 1% fungizone. Cells were then plated in 6-well plates, dispensing cells from approximately 1 eye cup per well. Cells were cultured until confluent after which the medium was changed to 1% FBS DMEM, 1% Penicillin/Streptomycin, 1% Gentamycin, and 1% fungizone.

### 2.4. Cell Transfection Methods

Peptides were transfected into cells using the delivery reagent PULSin (Polyplus, Illkirch-Graffenstaden, France). For immunofluorescence with cells cultured on coverslips in 48-well plates, transfection mixtures were prepared as follows: 0.5 µg of peptide was diluted in 50 µL of 20 mM Hepes Buffer (Polyplus) before the addition of 2 µL of PULSin. Transfection complexes were allowed to form for 15 min before. The mixture was then added to cells in serum-free DMEM for four hours before the medium was replaced with fresh 10% FBS in DMEM. For gene reporter assays in 96-well plates, the same protocol was followed but halving the transfection mixture (0.25 µg of peptide diluted in 25 µL of 20 mM Hepes Buffer with 1 µL of PULSin). For experiments with TAT-F1, TAT-GST, TAT-P5, and TAT-scramble, the cell medium was replaced with fresh medium, and the TAT peptides were added at the concentrations indicated in the figure legends. Sequences of the TAT-modified P5 and the scrambled negative control are provided in Table S2.

### 2.5. Cell Cytokine Treatment

Cells were serum-starved in a medium containing 1% FBS for 24 h before the addition of human recombinant TGFβ1 (PeproTech, London, UK). 20 ng/mL of TGFβ1 was used for IF and IB and 50 ng/mL for migration assays. Confluent HDMECs were incubated with 100 ng/mL LPS (Sigma) for 24 h. 

### 2.6. Immunoblotting

Cells were lysed and homogenized in 0.2 M Tris HCl pH 6.8, 6% SDS, 30% glycerol, 0.003% bromophenol blue, 100 mM DTT. Samples were cleared by centrifugation and run on SDS polyacrylamide gels at 25 mA constant current per gel. Gels were transferred onto nitrocellulose membranes using a wet transfer system (Bio-Rad, Hercules, CA, USA). Blots were blocked with 5% milk, 1× PBS, 0.1% Tween20 before primary antibody incubation overnight at 4 °C at the following dilutions: Mouse anti-αSMA (Sigma A2544), mouse anti-α-tubulin 1A2 [41], rabbit anti-cingulin (Santa Cruz sc-66831), mouse anti-GEF-H1 B4/7 [10], anti-JACOP (Abcam ab204500, Cambridge, UK), mouse anti-Vinculin (Sigma V9131), mouse anti-VSV [41], rabbit anti-ZO-1 [10], and mouse and rabbit anti-HA [42]. Membranes were washed in PBS, 0.1% Tween20 before secondary antibody incubation. Horseradish peroxidase-conjugated antibodies (Jackson ImmunoResearch, West Grove, PA, USA) were diluted in PBS, 0.1% Tween20, and incubated for 1 h at room temperature. Protein bands were visualized using chemiluminescence on high-performance chemiluminescence film (Hyperfilm Kodak, Rochester, NY, USA). Immunoblots were quantified using ImageJ by densitometry.

### 2.7. Immunofluorescence

Cells (3 × 10^4^) were seeded onto glass coverslips in a 48-well dish. For methanol fixation, cells were incubated in methanol at −20 °C for 5 min. Methanol was then replaced with PBS for 5 min, and cells were then blocked for 15 min with blocking buffer (1% BSA, 10% NaN_3_, 20 mM glycine, PBS). Alternatively, cells were fixed with 3% PFA in PBS for 15 min at room temperature and were then permeabilized with 0.3% Triton X-100, 1% BSA, PBS before blocking for 15 min with blocking buffer. Cells were stained with the following primary antibodies overnight at 4 °C in blocking buffer: mouse anti-αSMA (Sigma A2544), rabbit anti-cingulin (Santa Cruz sc-66831), mouse anti-GEF-H1 B4/7 [10], mouse anti-HA [42], rabbit anti-ICAM (Santa Cruz sc-7891), rabbit anti-JACOP (Abcam ab204500), mouse anti-Vinculin (Sigma V9131), mouse anti-VSV P5D4 [41], and rabbit anti-ZO-1 [10]. Cells were then washed twice with blocking buffer before secondary antibody addition. Secondary antibody incubation was carried out for 1 h at room temperature using FITC Donkey anti-mouse and Cy3 Donkey anti-rabbit IgG (Jackson Immunoresearch). For actin staining, fluorescently labeled Phalloidin (Sigma) was added with secondary antibodies. Cells were mounted on slides using Prolong Gold Antifade. Samples were analyzed using a Leica DMIRB microscope with a 63× NA1.4 Oil immersion lens. Cell area was calculated using ImageJ software by outlining cells stained for ZO-1 or F-actin using the polygon tool. The area of outlined shapes was then calculated. Values were normalized to control cells.

### 2.8. Gene Reporter Assays

HEK293 cells were seeded in triplicate or quadruplicate wells in 96-well plates the day before transfection. Cells were transfected with a plasmid containing an αSMA promoter driving firefly luciferase expression, a reference promoter driving renilla expression, and pCB6-GEF-H1-CA or empty vector (pCB6) using JetPei transfection reagent [17]. The next day luciferase values were measured using a dual luciferase assay kit (Promega Corp, Madison, WI, USA) using a FLUOstar OPTIMA microplate reader (BMG Labtech, Ortenberg, Germany). Values were normalized against cells transfected with GEF-H1-CA without inhibitor which was taken to be 1.

### 2.9. Migration Assay

Cells (3 × 10^4^) were seeded in 48-well dishes and serum-starved for 24 h. The cells were then treated with 50 ng/mL TGFβ1 and inhibitor for 2 days. Monolayers were then scratched with a yellow pipette tip to create a wound. Bright-field images were taken at 0 h and 16 h using an epifluorescent microscope. Images were quantified by measuring the area of wounds at 0 and 16 h using ImageJ software. The percentage of wound closure was calculated using ImageJ. Migration of MDA-MB-231 on Matrigel-coated dishes was performed as described previously, recording the cells for 4 h by phase-contrast microscopy at 37 °C [43].

### 2.10. Paracellular Permeability

HDMECs were seeded on Transwell filters in triplicate (6.5 mm diameter, 0.4 μM pore size, Corning) coated with Matrigel (Corning Inc., New York, NY, USA). Endothelial paracellular permeability was assayed by adding 4kD FITC- and 70 kD Rhodamine-conjugated dextran (1 mg/mL final concentration, Sigma-Aldrich), to the apical side. Permeability was determined by measuring the respective fluorescence intensity that was emitted from 25 µL of medium taken from the basolateral side 3 h later using a FLUOstar OPTIMA microplate reader (BMG Labtech). Values were then normalized against filters without cells.

### 2.11. Toxicity Assays

HEK293 and MDCK cells were seeded in triplicate wells and incubated overnight with the indicated concentration of TAT-P5 or TAT-F1. The next day 30 μL of medium was removed from each well and incubated with 30μL of CytoTox-ONE (Promega) reagent for 40 min. Fluorescence was then measured to assess LDH release. To assess Caspase 3/7 activity cells were assayed using Apo-ONE Homogenous Caspase-3/7 assay (Promega) according to manufactures instructions.

### 2.12. Experimental Autoimmune Uveitis (EAU)

Animal experiments were conducted according to UK Home Office Regulations, the UK Animals (Scientific Procedures) Act of 1986, and approved by the Institute’s Animal Welfare and Ethical Review Body (P2C479FB6). C57BL/6J mice were obtained from Charles River UK and housed in vented cages with water and food ad libitum. EAU was induced as previously described [44]. In brief, 6–8 week-old animals were immunized by subcutaneously injecting 400 µg interphotoreceptor retinoid-binding protein (IRBP)1-20 (GPTHLFQPSLVLDMAKVLLD, Generon, Slough, UK) in PBS/DMSO emulsified with Complete Freud’s Adjuvant (CFA, Sigma-Aldrich, Gillingham, UK) supplemented with Mycobacterium tuberculosis (final 2.5 mg/mL, Difco, Voigt Global Distribution). Additionally, mice received an intraperitoneal injection of 0.4 mg pertussis toxin (Sigma-Aldrich, Gillingham, UK) in 0.1% mouse serum/RPMI. Sham mice received a vehicle injection (PBS) emulsified with CFA without IRBP. Disease was monitored by weekly fundoscopy (Micron III, Phoenix Technology Group, Pleasanton, CA, USA) starting day 14 post-immunization. Disease progression was then scored using the fundoscopy images as previously detailed by Xu et al. [45] and Copland et al. [46]. Briefly, four different parameters (optic disc neuropathy, vasculitis, retinitis, and structural damage) were clinically scored, each from 0 (no disease) to 5 (severe disease) for each eye. The criteria for each score in each of the categories were as defined by Copland et al. [46]. After assessing each individual parameter, the total clinical score (sum of all parameters) was computed and disease progression across the time course was calculated. Clinical scoring was performed by two researchers independently on blinded images. After initial scoring at day 14, animals with EAU were divided into treatment groups so that each group had the same number of animals with the same disease severity. Animals of different treatment groups were mixed within cages, and male and female animals were evenly spread across treatment groups within this study. No sex-based differences were observed. Mice with a clinical score of ≥ 10 at day 14 were excluded from the study since established structural damage is not reversible. Based on the in vitro work, the concentration of the inhibitor was chosen in pilot studies. Treatment with 0.04 mg/mL TAT-P5 commenced on day 15 after disease onset was confirmed. Daily eye drops of 6 µL were carefully applied while restraining the mice manually for 30 sec before releasing them back into the cage. PBS eye drops were used as vehicle controls for the inhibitor and mice with comparable initial disease on day 14 were assessed. On days 29 or 36, the mice were sacrificed, retinas were collected for flow cytometry or whole eyes were enucleated for histology. For weekly fundoscopy, mice were anesthetized by inhalation of 1% isoflurane followed by fluorescent angiography. For angiographies, 0.1% Fluorescein/PBS was injected subcutaneously after fundoscopy and two images were obtained, after 1.5 min and 7 min. Images were quantified as previously described using ImageJ [47]. If SD-OCT was performed prior to fundoscopy and angiography, ketamine (70 mg/kg) and metetomidine (1 mg/kg) anesthesia was used. For imaging, pupils were dilated with one drop of 1.0% tropicamide after the anesthesia and carbomer eye gel (0.2%) was used to lubricate the corneal surface. SD-OCT imaging was conducted using the Envisu 2200 equipped with a mouse retina lens and Vivo Vue, version 2.0, software. SD-OCT images were quantified for vitreous infiltrate using The Iowa Reference Algorithms (Retinal Image Analysis Lab, Iowa Institute for Biomedical Imaging, Iowa City, IA, USA) and ImageJ [15,48,49,50]. Per eye 29 images centered around the optic nerve head were quantified and the average number of infiltrates was calculated for each analyzed eye.

### 2.13. Flow Cytometry

Retinas were collected and cultured as single-cell suspension for 4 h in 10% FBS/RPMI 1640 supplemented with a cell stimulation cocktail (1:500, eBioscience 00-4975-03). For staining, whole single retinas were collected, washed in PBS and stained with ZOMBIE-NIR viability dye (1:100 in PBS, BioLegend 423106) at RT for 15 min to label dead cells. Subsequently, cells were washed in Flow Cytometry Staining Buffer (eBioscience 00-4222-26) and extracellular antigens were stained with respective antibodies (1:50, BioLegend 100210, 102049, 103150; BD-Horizon 563790) for 30 min on ice. Following a washing step with FC buffer (400× *g*, 5 min, 4 °C), cells were fixed in 1× FOXP3 Fix/Perm buffer/PBS (BioLegend) for 20 min, washed, and permeabilized in 1× FOXP3 Perm buffer/PBS (BioLegend) at RT for 15 min. Intracellular antibodies (BioLegend 505810 and 126419) were stained after another wash with antibodies diluted 1:50 for 30 min at room temperature in the dark. Excess antibody was removed by washing again and cells were resuspended in 300 µL of the staining buffer prior to overnight storage at 4 °C. Cells were analyzed using a BD Fortessa X-20 flow cytometer, channel compensation was conducted using OneComp eBeads compensation beads (Invitrogen 01-1111) according to the manufacturer’s protocol. Data were analyzed using FlowJo (BD, Wokingham Berkshire, UK). Debris and dead cells (ZOMBIE-NIR positive) were excluded from analysis, prior to the identification of CD45^+^ cells. Subsequent gating was conducted on CD3+ cells (T cells), either CD3^+^CD4^+^ or CD3^+^CD4^+^. The latter population was investigated using specific T cell subset markers (IFNy^+^, IL-17^+^, FOXP3^+^, CD25^high^).

### 2.14. Histology

For histological assessment, mice were killed by cervical dislocation 28 days after EAU induction. Eyes were enucleated and fixed in 4% glutaraldehyde/PBS for 1 h and subsequently in 4% PFA for 16 h prior to paraffin embedding to retain tissue integrity. 10 µm-sections were stained with hematoxylin and eosin using an Autostainer (Leica, Wetzlar, Germany) and whole sections in the optic nerve plane were scored on a scale of 0 to 4 as previously described [44,51]. In brief, 0, no disease and normal retinal architecture; 0.5, mild inflammatory cell infiltration and no tissue damage; 1, mild infiltration in the vitreous, uvea, and retina, with the presence of retinal folds, vasculitis, and one small granuloma; 2, moderate infiltration of the uvea, vitreous, and the retina, presence of retinal folds, small granulomas, vasculitis, focal shallow detachments, and focal photoreceptor cell damage; 3, moderate to severe infiltration in the vitreous, retina and uvea, and the presence of extensive retinal folding with large detachments, moderate photoreceptor cell damage, subretinal neovascularization, and medium-size granuloma lesions; 4, severe infiltration, diffuse retinal detachment, subretinal neovascularization, hemorrhage, extensive photoreceptor cell damage, and large granuloma lesions. For immunofluorescence staining, sections were de-paraffined and rehydrated in a descending ethanol row prior to antigen retrieval through incubation with 0.02 mg/mL proteinase K (Sigma) for 4 min. Sections were washed twice with PBST (0.05% Tween 20/PBS) before being permeabilized with 1% Triton X100/PBS for 15 min. Following three washes with PBST of 15 min each, sections were blocked for 1 h (2% donkey serum/1% BSA/0.1% Triton X100/0.05% Tween 20/PBS). Primary antibody against GFAP (Dako Z0334) in 1:10 pre-diluted blocking buffer was incubated at 4 °C overnight. After washing trice with PBST, sections were incubated with secondary antibody plus Hoechst at room temperature for 1 h. Sections were washed trice in PBST and mounted using Prolong. GFAP-positive areas and total retinal areas were measured using thresholding (ImageJ). All histological quantifications were performed on blinded samples.

### 2.15. Statistical Methods

Statistical analysis and dose-response curve modeling for IC50 determinations were performed with JMP Pro 15 or GraphPad Prism. Statistical significance was analyzed as indicated in the legends, performing either Student’s t- and, for multiple comparisons, ANOVA tests, or Mann-Whitney and Wilcoxon tests for nonparametric data analysis. No samples were excluded from the analysis. Sample sizes of animal experiments were based on variability observed in previous studies and expectations of an effect size of at least 25%.

## 3. Results

### 3.1. Identification of Peptide Inhibitors of GEF-H1

GEF-H1 contains a C1 domain, which binds microtubules, and the Dbl-homology (DH) and pleckstrin-homology (PH) module that binds to RhoA and catalyzes the exchange of GDP for GTP on RhoA (Figure 1A). GEF-H1 also possesses a C-terminal domain that contains an autoinhibitory domain (CTD) [17]. Removal of the C1 and CTD domains leads to a constitutively active mutant that is neither sequestered to microtubules by the C1 domain nor autoinhibited by the CTD (Figure 1A) [17]. We employed two approaches to identify inhibitors for GEF-H1. The first approach was based on identifying the minimal sequence required for autoinhibition by the CTD. The CTD was hence divided into four fragments, F1-F4. F1, F2, and F3 were generated as GST-fusion proteins and further assayed for their inhibitory activity on GEF-H1 functions. F4 could not be expressed as a recombinant protein and was therefore omitted from further experiments (Figure 1A). The second approach was based on structural modeling of the GEF-H1/RhoA complex and designing peptides likely to inhibit the formation of the complex. The prediction was based on structures of homologous proteins in complex with RhoA, as GEF-H1 has not yet been crystallized. There are several known crystal structures of other GEFs that can form GEF/RhoA complexes. We used the X-ray crystal structure (PDB: 1XCG) of the DH/PH module of PDZRhoGEF crystallized with nucleotide-free RhoA [52], the form of RhoA that binds tightly to GEFs, and overlaid it with the sequence of the DH/PH module of GEF-H1 (Figure 1B). Regions likely to represent areas of interaction between the DH domain of GEF-H1 and RhoA were mapped and used to design five peptides (P1, P2, P3, P4, and P5) that may interact with GEF-H1 (Figure 1C). P1 and P2 represented the same region but P2 contained a V to D substitution to increase polarity and stabilize the interaction with a His residue in GEF-H1. P4 and P5 also targeted the same region but P5 was an extended form of P4 to stabilize the α-helical structure and contained an L to A substitution to reduce potential steric clashes within the GEF-H1 DH domain.

**Figure 2:**
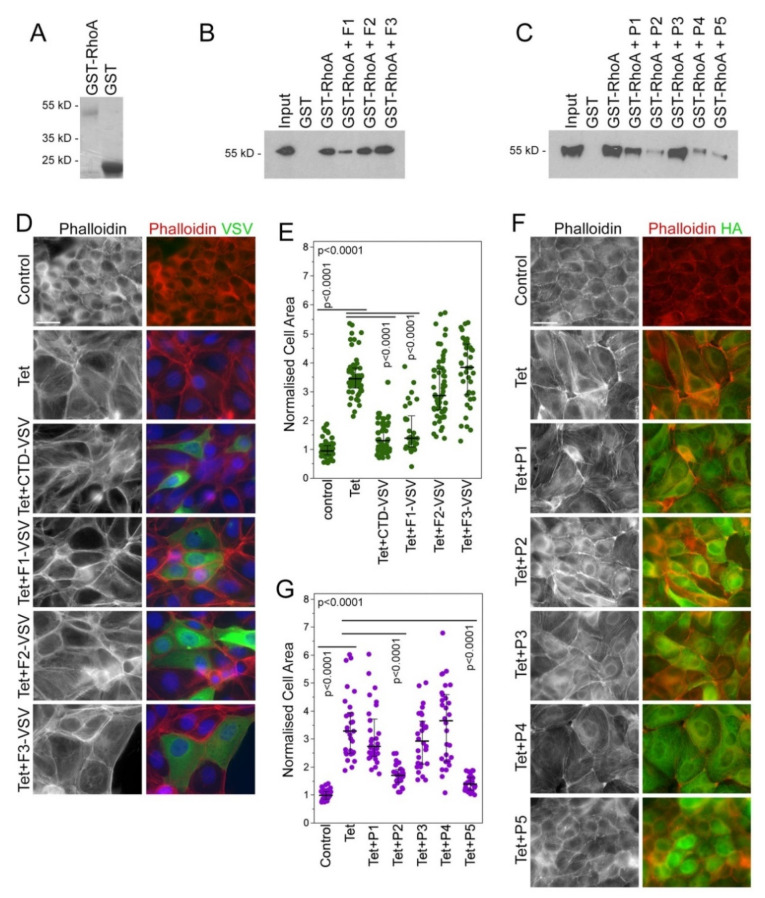
Selection of GEF-H1 inhibitors that inhibit RhoA binding and GEF-H1 overexpression phenotypes. (**A**–**C**) Recombinant fusion proteins of GST and RhoA were used for pulldown of constitutively active GEF-H1 from cell extracts. Candidate fragments and peptide inhibitors were added to the pulldown. Recombinant fragments from the CTD of GEF-H1 (**B**) or peptides (**C**) were added. Note, F1 and P2, P4 and P5 peptide inhibitors block the interaction between RhoA and active GEF-H1. (**D**–**G**) A stable cell line for tetracycline (Tet)-inducible expression of HA-tagged GEF-H1 was transiently transfected with plasmids encoding VSV-tagged CTD fragments of GEF-H1 (**D**) or candidate peptide inhibitors (**F**). The cells were then fixed and stained for either F-actin with fluorescently labeled phalloidin and the VSV-epitope to detect cells expressing the VSV-tagged CTD fragments (**D**) or fluorescently labeled phalloidin and the HA-epitope to reveal expression of HA-tagged GEF-H1 (**F**). Cell areas were then quantified as a measure of cell spreading, which increases in response to GEF-H1 overexpression (**E**,**G**). The quantifications show values for cell sizes measured over 2 (**E**) or 3 (**G**) experiments. P-values were calculated with Wilcoxon and Mann-Whitney tests. Scale bars, 20 μm.

The inhibitors were tested in in vitro assays for their ability to inhibit GEF-H1-RhoA binding and GEF-H-induced cell morphology changes driven by stress fiber formation and transcriptional activity. As the DH/PH module of GEF-H1 needs to bind to RhoA to catalyze the exchange of GDP for GTP, we first tested whether the candidate GEF-H1 inhibitors can inhibit the binding of nucleotide-free RhoA. Immobilized GST-RhoA was used to pulldown constitutively active GEF-H1 from MDCK cell extracts. Cell extracts were then incubated with 100 µM of the different candidate inhibitors before pulldown. The CTD-derived inhibitor F1, as well as the peptide inhibitors P2, P4, and P5, reduced GEF-H1-RhoA binding (Figure 2A–C), indicating that they interfere with the binding of GEF-H1 to its substrate RhoA.

RhoA activation by GEF-H1 regulates the cytoskeleton; thus, we used a stable MDCK cell line with a tetracycline (Tet) inducible expression of HA-tagged GEF-H1 to assess the inhibitors in biological assays. Cells were transiently transfected with plasmids encoding VSV-tagged CTD fragments of GEF-H1 (F1–F3) or by peptide transfection of P1–P5 to test whether inhibitors can reduce GEF-H1 induced changes to the actin cytoskeleton and cell morphology. MDCK cells overexpressing GEF-H1 exhibit increased stress fiber formation and adopt an extended, flatter morphology. Cell areas were quantified to measure inhibition of GEF-H1-induced cell spreading (Figure 2D,E, CTD fragments; Figure 2F,G, peptides). Transfection of F1, P2, and P5 reduced stress fiber formation and cell spreading. Quantification showed that cells transfected with F1 and P5 had the greatest reduction in GEF-H1 induced cell spreading. In contrast to P5, P4 was not effective in cells, suggesting that the extension of the peptide increased helical stability or improved permeability. Based on this initial characterization, we selected P5 and F1 for further development and validation in disease models.

### 3.2. Characterization of Membrane-Permeable GEF-H1 Inhibitors

**Figure 3:**
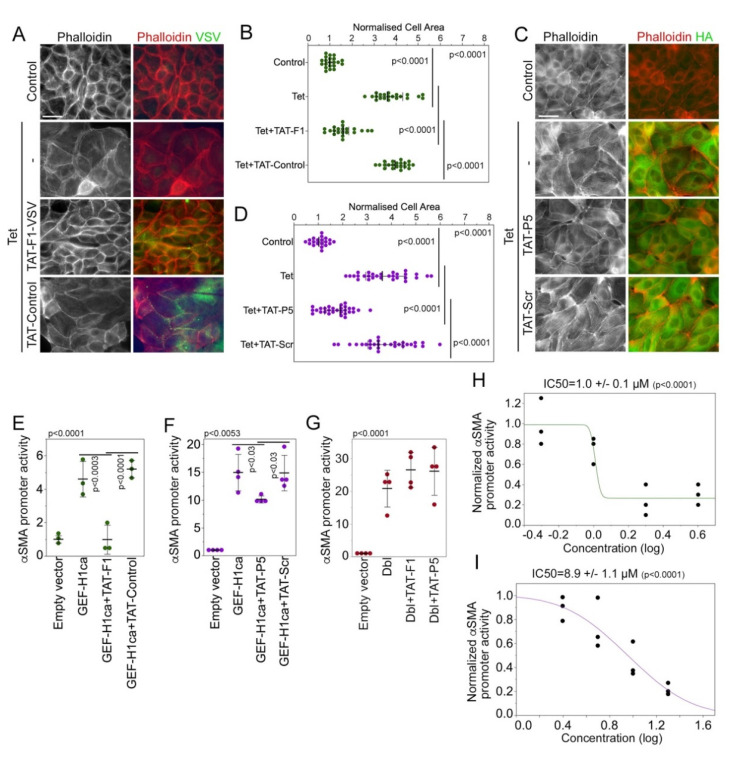
Membrane-permeable GEF-H1 inhibitors impede overexpressed GEF-H1. (**A**–**D**) TAT-F1 or TAT-Control (**A**,**B**: 4 µM), or TAT-P5 or TAT-Scr (**C**,**D**: 20 µM) were incubated overnight with MDCK cells induced to overexpress HA-GEF-H1. The cells were then analyzed by staining for F-actin and the VSV-epitope to detect cells expressing the VSV-tagged CTD fragments (**A**) or F-actin and the HA-epitope to monitor HA-tagged GEF-H1 expression (**C**). Cell areas were measured to assess cell spreading (**B**,**D**). The quantifications show values for cell sizes measured over 2 (**B**) or 3 (**D**) experiments. *p*-values were calculated with Wilcoxon and Mann-Whitney tests. (**E**–**I**) HEK 293 cells were co-transfected with a plasmid containing an αSMA promoter driving firefly luciferase expression, one with a CMV promoter stimulating renilla luciferase expression, and a plasmid encoding GEF-H1-CA. The cells were incubated with TAT-F1 (**E**,**G** 4 µM) or TAT-P5 (**F**,**G** 20 µM) or with the concentrations indicated (**H**, TAT-F1; **I**, TAT-P5) overnight. The activity of the luciferases was then measured and the activity of the αSMA promoter was expressed as a ratio between firefly and renilla luciferase activity. Shown are independent determinations, and *p*-values derived from ANOVA and two-sided *t*-tests. Scale bars, 20 μm.

We next generated membrane-permeable variants of F1 and P5 by attaching the TAT sequence, a cell-penetrating peptide. The addition of TAT-motifs is a common strategy to convert peptides and proteins to variants that are more membrane-permeable and is used for compounds already in clinical trials [53]. TAT-F1 was expressed and purified as a GST-tagged fusion protein. TAT-P5 and a scrambled version (TAT-scr) were synthesized. TAT-F1 and TAT-P5 were then assessed for their ability to suppress GEF-H1-induced cytoskeletal rearrangements in MDCK cell lines. Both TAT-F1 and TAT-P5 reduced stress fiber formation and cell spreading in MDCK cells overexpressing HA-tagged GEF-H1, whereas the respective control protein or peptide did not detectably affect these parameters (Figure 3A–D).

GEF-H1 regulates αSMA expression and promoter activity in a RhoA-dependent manner [17]. Therefore, we next tested whether the inhibitors can attenuate GEF-H1-induced activation of the αSMA promoter using a luciferase reporter gene assay [17]. Both TAT-F1 and TAT-P5 inhibited GEF-H1-mediated αSMA promotor stimulation in a dose-dependent manner with IC50s of 1 and 9 µM, respectively (Figure 3E,F,H,I). TAT-GST or TAT-scr did not affect GEF-H1-induced αSMA promoter activity. Thus, TAT-F1 and TAT-P5 inhibit GEF-H1-regulated activity of the αSMA promoter.

We next tested the specificity of the inhibitors for GEF-H1. ARHGEF18/p114RhoGEF is a RhoA GEF that, like GEF-H1, binds via its PH domain to cingulin and is evolutionarily closely related to GEF-H1 [28]. Its DH domain is hence highly homologous to the one of GEF-H1. In contrast to overexpression of GEF-H1, induction of p114RhoGEF expression in a tetracycline-regulated MDCK cell line induces cell rounding as it stimulates RhoA and myosin-II activation at the junctional complex [28]. Neither TAT-F1 nor TAT-P5 had any apparent impact on p114RhoGEF-induced morphological changes (Figure S1). Similarly, the GEF Dbl/MCF2, which gave its name to the Dbl homology domain, induces increased αSMA promoter activity in a transient transfection assay; however, neither TAT-F1 nor TAT-P5 inhibited Dbl-induced αSMA promoter stimulation (Figure 3G). Thus, TAT-F1 and TAT-P5 show specificity for GEF-H1 even if compared to GEFs with highly homologous DH domains.

We next tested whether the inhibitors are toxic to cultured cells. MDCK and HEK293 cells were incubated with increasing concentrations of either TAT-F1 or TAT-P5 overnight before analysis of LDH release as a measure of necrosis or Caspase 3/7 activity to assess induction of apoptosis (Figure S2). Neither assay revealed an increase in cytotoxicity in response to inhibitor treatment. Thus, TAT-F1 and TAT-P5 inhibit GEF-H1-induced RhoA signaling without inducing cytotoxic effects.

### 3.3. GEF-H1 Inhibitors Antagonize TGFβ and LPS Signaling in Cellular Disease Models

**Figure 4:**
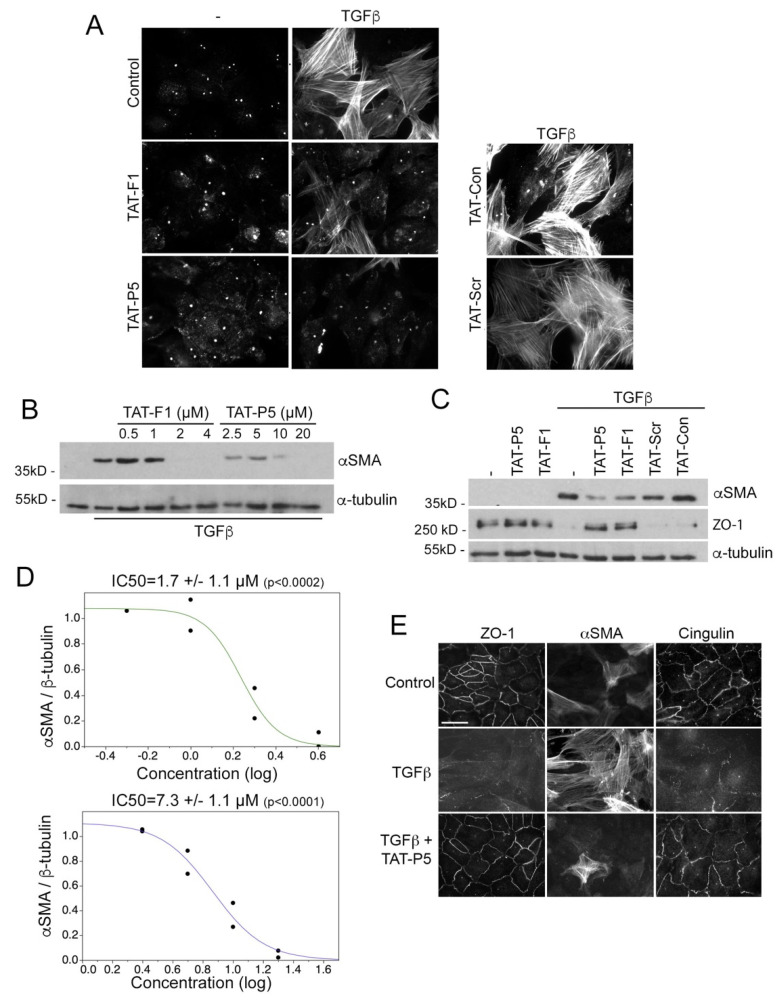
GEF-H1 antagonists inhibit epithelial mesenchymal transition induced by TGFβ in primary retinal pigment epithelial (RPE) cells. (**A**–**D**) Primary RPE cells were incubated without or with TGFβ for five days in the presence of TAT-F1, TAT-P5, TAT-scr, or TAT-control expression of αSMA positive cells was analyzed by immunofluorescence (**A**) or immunoblotting (**B**–**D**; α-tubulin was used as a loading control). The graphs in panel D show quantifications of concentration-dependent expression of αSMA normalized by α-tubulin expression derived from densitometric scanning of the immunoblots. Note, that GEF-H1 inhibitors strongly reduce TGFβ-induced αSMA expression in a concentration-dependent manner (TAT-F1, upper and TAT-P5, lower graph). Panel C also shows immunoblots for ZO-1, revealing attenuation of ZO-1 downregulation by GEF-H1 inhibitors. (**E**) Expression of the junctional markers ZO-1 and cingulin, as well as αSMA was analyzed by immunofluorescence after incubating without inhibitors or with either TAT-P5. Note, TAT-P5 inhibits TGFβ induced αSMA expression and loss of junctional ZO-1 and cingulin staining. Scale bar, 30 μm.

**Figure 5:**
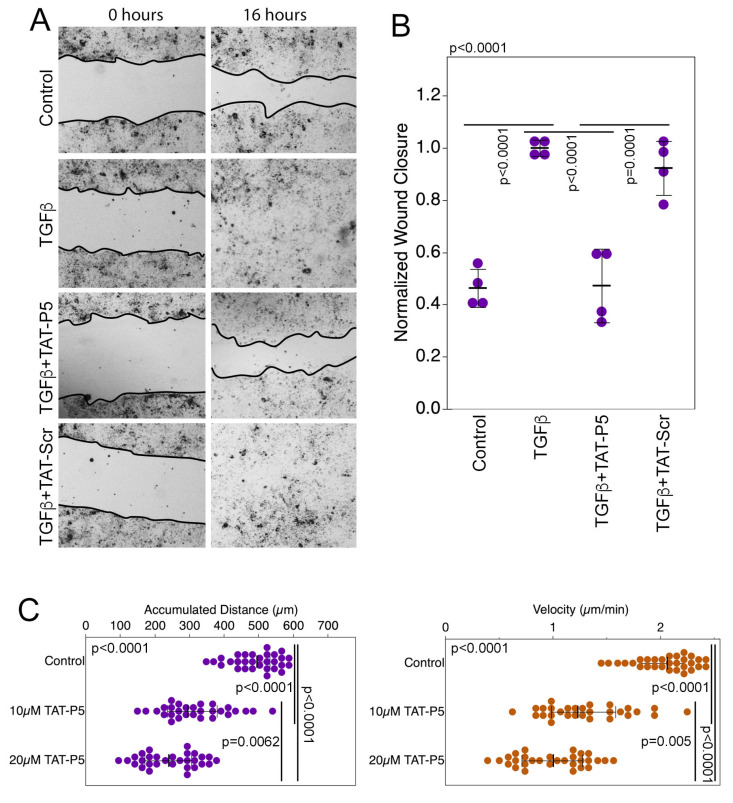
TAT-P5 inhibits RPE and MDA-MB-231 cancer cell migration. (**A**,**B**) RPE cells without or with TAT-P5 were assayed for cell migration without or with TGFβ stimulation using a scratch wound assay. The quantification in panel B shows values from independent determinations. *p*-values are derived from ANOVA and *t*-tests. (**C**) GEF-H1 inhibitors attenuate distance and velocity of MDA-MB-231 breast cancer cell migration on Matrigel-coated tissue culture plates. Shown are individually tracked cells derived from three different cultures. *p*-values are derived from Wilcoxon and Mann-Whitney tests.

GEF-H1 functions as an effector of TGFβ during induction of RPE cell EMT and migration, which is a major component of different retinal degenerative diseases and leads to retinal detachments in proliferative vitreoretinopathy [17]. This can be recapitulated with primary cultures of porcine RPE cells that induce αSMA expression, loss of cell-cell junctions, and increased migration in response to TGFβ, which is dependent on GEF-H1 [17]. Both TAT-F1 and TAT-P5 inhibited TGFβ-induced αSMA expression in RPE cells, whilst TAT-GST or TAT-scr did not (Figure 4A–C). Inhibition of αSMA expression was concentration-dependent with IC50 values comparable to those observed for ectopic GEF-H1 inhibition in the αSMA reporter gene assays (Figure 4D). TAT-P5 and TAT-F1 also inhibited the downregulation of the tight junction protein ZO-1 (Figure 4C,E). To assess the effect of inhibitors on cell migration, confluent TGFβ-stimulated cells were analyzed with a scratch assay. Monolayers were scratched and migration was followed in the presence or absence of inhibitors. TAT-P5 reduced scratch closure from 100% to 59% over a period of 16 h, compared to TAT-scr, which had no effect (Figure 5A,B). TAT-F1 did not consistently reduce cell migration in TGFβ-stimulated RPE cells (Figure S3). Thus, TAT-P5 prevents TGFβ-induced RPE epithelial to mesenchymal transition by inhibiting αSMA induction, downregulation of junctional proteins, and cell migration.

Increased migration is a key feature of tumor cells; hence, we further assessed whether TAT-P5 blocks migration of the invasive breast cancer cell line MDA-MB-231. GEF-H1 and RhoA are required for the migration and invasion of MDA-MB-231 cells [54]. Cells were cultured on extracellular matrix-coated tissue culture plates. Migration was then analyzed by live-cell microscopy for 4 h to determine the effect of TAT-P5. The inhibitor reduced velocity and overall distance of migration (Figure 5C). Hence, GEF-H1 inhibitor TAT-P5 inhibits the migration of metastatic tumor cells.

**Figure 6:**
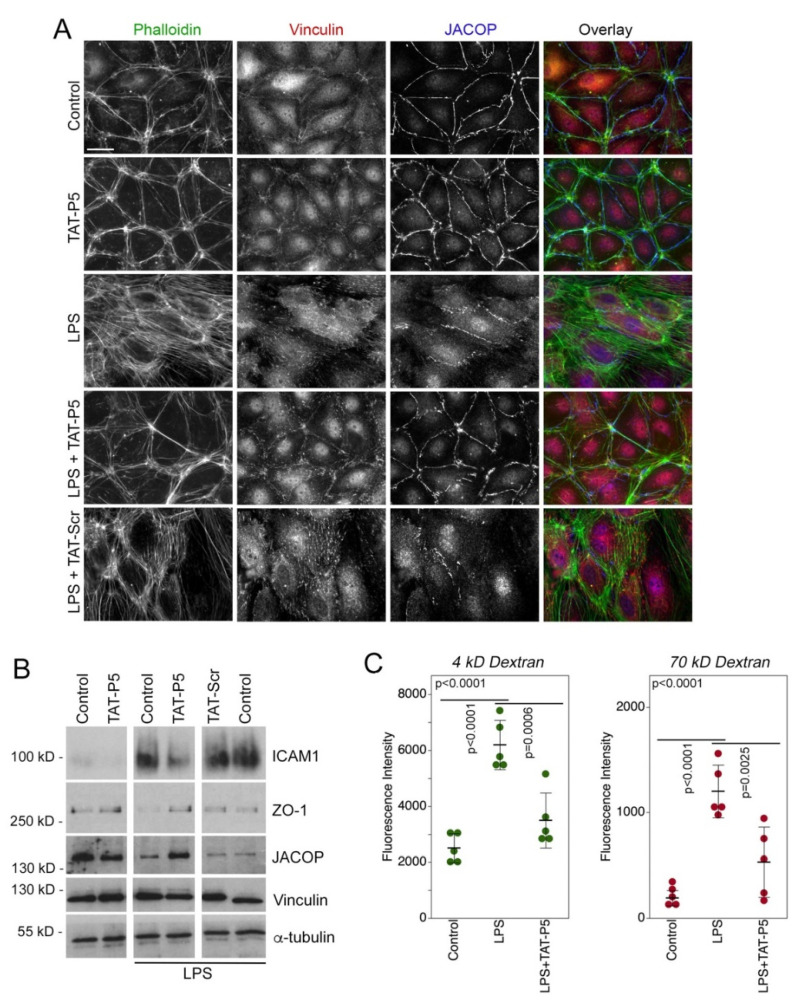
GEF-H1 inhibitors block endothelial inflammatory responses induced by LPS. Human primary endothelial cells were stimulated with LPS in the presence or absence of the indicated GEF-H1 inhibitors. Cells were then analyzed by immunofluorescence (**A**, F-actin, vinculin, and JACOP localization) and (**B**) immunoblotting for expression of ICAM1, a protein upregulated by inflammatory stimuli; ZO-1; JACOP, vinculin, and α-tubulin as a loading control. (**C**) Quantification of paracellular permeability for 4 and 70 kD fluorescent dextrans in similar experimental conditions is shown. Shown are independent determinations. *p*-values are derived from ANOVA and *t*-tests. Note: TAT-P5 and TAT-F1 inhibit cytoskeletal remodeling, ICAM1 induction, and barrier permeability increased induced by LPS. Scale bar, 20 μm.

LPS is a key trigger of inflammatory phenotypes in endothelial cells and stimulates GEF-H1 activation and contributes to TLR signaling [39,55,56]. We next used human primary microvascular endothelial cells (HDMECs) to assess whether membrane-permeable GEF-H1 inhibitor TAT-P5 can rescue cytoskeletal remodeling and paracellular leakage induced by LPS. Cells were stimulated with 100 ng/mL LPS for 24 h in the presence or absence of TAT-P5. Immunofluorescence analysis showed that LPS stimulation of HDMECs resulted in remodeling of the actin cytoskeleton, increased focal adhesion formation, and disruption of the localization of JACOP, a cell-cell junction protein. LPS-stimulated cells incubated with TAT-P5 had reduced stress fiber formation, focal adhesion formation, and junctional disruption compared to those incubated with TAT-Scr (Figure 6A). Immunoblotting showed that LPS-induced reduced expression of junctional proteins and increased expression of ICAM1 were inhibited by TAT-P5 (Figure 6B). Consequently, increased paracellular permeability induced by LPS was rescued by TAT-P5 (Figure 6C). Thus, TAT-P5 inhibits LPS-induced cytoskeletal remodeling, ICAM1 induction, and loss of barrier function in microvascular endothelial cells. Similarly, TAT-P5 also prevented reorganization of the cytoskeleton and junction disruption induced by thrombin (Figure S4).

### 3.4. GEF-H1 Inhibitors Ameliorate Retinal Autoimmune Disease

GEF-H1 inhibitor TAT-P5 inhibited fibrotic processes and proinflammatory signaling-induced barrier loss in vitro; hence, we next tested whether the inhibitor can attenuate inflammatory disease in vivo using the experimental autoimmune uveitis (EAU) mouse model. EAU is an autoimmune inflammatory disease affecting the neural retina and involves the upregulation of cytokines such as TNFα [57,58,59,60]. Expression of GEF-H1 is induced in patient RPE in response to uveitis and other retinal insults [17].

**Figure 7:**
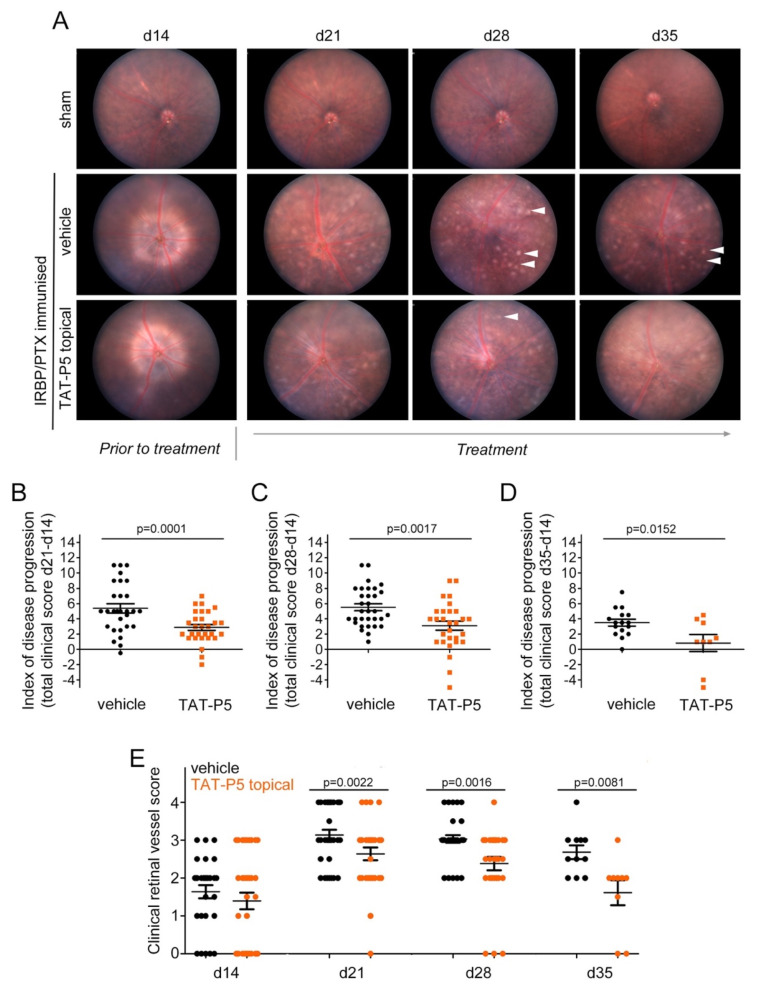
GEF-H1 antagonist TAT-P5 inhibits progression of EAU. EAU was induced in mice and induction of disease was evaluated at day 14 post-immunization. On day 15, daily topical eye drop treatment with 6μL TAT-P5 (0.04 μg/μL) or saline solution (vehicle control) commenced (16 immunized mice were used for each type of treatment over 5 experiments). Disease progression was assessed by fundoscopy every seven days until days 28 or 35 (**A**). Fundus images were scored as described in Material and Methods, and the index of disease as a change from day 14 to 21 (**B**, n = 29 eyes per condition), to 28 (**C**, n = 32 and 29 eyes per condition, respectively) or to day 35 (**D**, n = 16 and 9 eyes, respectively) was calculated. At day 14, the total clinical score was <10 for all mice included in the study. (**E**) Clinical vessel scoring as one of the four parameters of the fundus scoring applied. For the breakdown of the other clinical parameters, see Figure S4. Statistical significance was assessed using *t*-tests. Indicated are means ±SEM. ANOVA tests resulted in *p* < 0.0001 for the time course of disease index (shown as separate panels in **B**–**D**) and for the retinal vessel score time course (panel **E**). Sham immunized mice served as negative controls. Arrowheads indicate immune cell infiltrates.

EAU was induced in wildtype C57BL/6J mice using a peptide derived from interphotoreceptor retinoid-binding protein (IRBP) and disease manifestation was assessed after 14 days post-immunization (d14) prior to starting the treatment. Mice were then treated with 0.04 mg/mL TAT-P5 applied as daily eye drops and disease progression was monitored weekly by fundoscopy until day 35 (Figure 7A). Clinical scores were calculated for each eye receiving either vehicle treatment or inhibitor treatment and the index of disease was calculated as a measure of disease progression (Figure 7B–D). The clinical score is the sum of individual assessments made for optic disc swelling, tissue infiltrates, blood vessels with clustered infiltrates of immune cells, and structural damage (Figure 7E and Figure S5). The clinical scores revealed a suppressive effect of the inhibitor already after one week of treatment, day 21 (Figure 7B), a time point when disease usually peaks in this model. This positive effect persisted through to day 28 (Figure 7C) and day 35 (Figure 7D). The different clinical parameters considered individually all indicated reductions in disease except for structural damage of which we observed only little in non-treated animals (Figure 7E and Figure S5). Especially, the parameters reflecting immune cell infiltration—retinal infiltration and blood vessels—were positively affected by the TAT-P5 treatment. Fluorescence fundus angiography further revealed improved blood/retinal barrier integrity in eyes treated topically with TAT-P5 (Figure S6). Thus, TAT-P5 inhibits inflammation and blood vessel leakage in a mouse model of autoimmune disease if applied after disease was clinically apparent.

**Figure 8:**
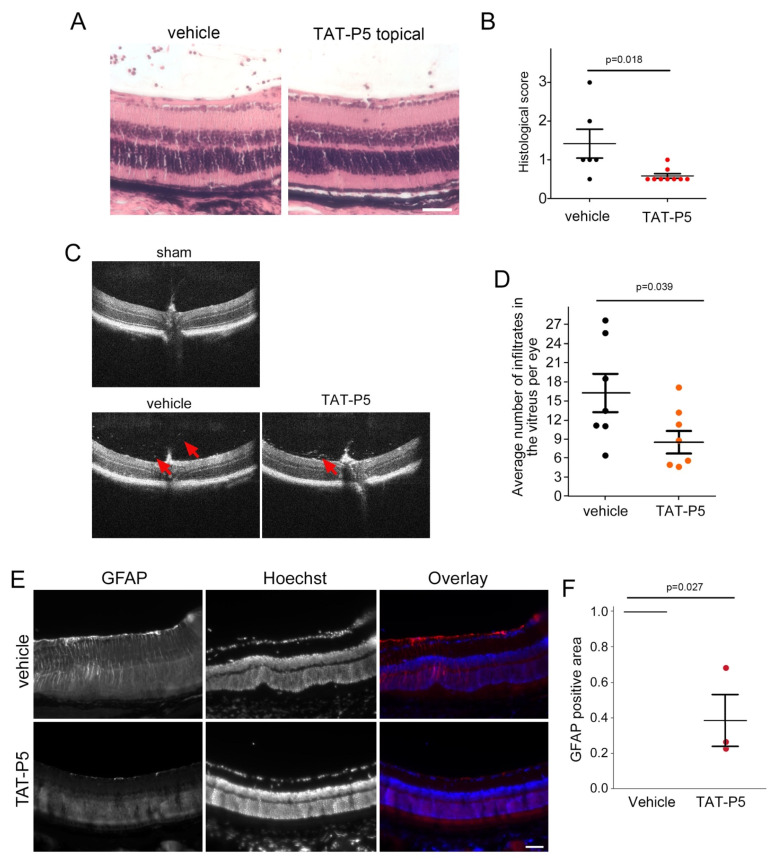
TAT-P5 reduces retinal damage in EAU. EAU was induced in mice and treated and analyzed as described in Figure 7A,B H&E-stained paraffin sections from vehicle control and TAT-P5 eyes were analyzed at day 29 (**A**). The eyes were histologically scored upon inspection of entire retinal sections (**B**, see also Figure S8A). (**C**,**D**) SD-OCT images obtained on day 28 after disease induction were quantified for vitreous infiltrates. 29 images of the central region around the optic nerve were quantified per eye and averaged to obtain a single value for each diseased eye. Arrows indicate infiltrates. (**E**,**F**) Retinal sections were stained for GFAP to label activated Müller glial cells. GFAP staining intensity was analyzed in sections derived from 3 eyes for each condition (see also Figure S8B). Vehicle-treated sections were set to 1 for each pair analyzed, which is indicated with a line that represents the three controls. Each data point represents one eye and statistical significance was assessed using two-sided (**C**,**D**) or one-sided single sample (F, comparing to a test mean of 1) tests. Scale bars indicate 50 µm.

We next analyzed H&E-stained paraffin sections from EAU eyes treated with either the vehicle control or TAT-P5 until day 29. This revealed that TAT-P5 treated eyes had significantly less damage and less vitreal immune cell infiltration as quantified with a histological score compared to vehicle controls (Figure 8A,B and Figure S8A). Positive attenuation of vitreal immune cell infiltration by TAT-P5 was further confirmed by spectral domain-ocular coherence tomography (SD-OCT) at day 28 (Figure 8C,D). Although TAT-P5 treated eyes showed an overall reduction in immune cell infiltrate, the relative size of different immune cell populations was not affected as analyzed by flow cytometry (Figure S7).

During IRBP peptide-induced EAU progression, changes in retinal histology correlate with GFAP expression by Müller cells and loss of retinal function [61,62]. Figure 8E,F and Figure S8B show that induction of GFAP was inhibited by TAT-P5. These data indicate that the TAT-P5 GEF-H1 antagonists inhibit the induction of retinal histological damage and Müller cell gliosis in a mouse model of retinal disease.

## 4. Discussion

GEF-H1/ARHGEF-2 is activated in inflammation and fibrosis to drive signaling mechanisms leading to epithelial and endothelial barrier failure, EMT, tissue degeneration, and cell migration. We have generated GEF-H1 inhibitors that specifically block signaling by GEF-H1 and successfully tested the best of them, TAT-P5, in in vitro disease models for inflammatory and fibrotic diseases. In an in vivo experimental autoimmune uveitis disease mouse model, TAT-P5 inhibited disease progression when animals were treated after disease onset. Thus, GEF-H1 can be targeted therapeutically, and its inhibition is sufficient to ameliorate disease outcomes. The newly developed inhibitors, therefore, represent an important milestone toward the therapeutic inhibition of GEF-H1 and establish a new therapeutic approach. Given the central role of GEF-H1-regulated processes such as fibrosis, inflammation, and cell migration in common and still difficult to treat diseases, therapeutic GEF-H1 inhibition may be beneficial to a wide range of clinically relevant and widespread diseases that are still difficult to treat.

GEF-H1 is regulated by different mechanisms ranging from microtubule binding and junctional recruitment to MAP kinase phosphorylation and increased gene expression [29,38]. The newly developed inhibitors target RhoA activation; hence, they are likely to be independent of the mode of GEF-H1 stimulation. We identified two different types of GEF-H1 inhibitors. P5 was identified based on modeling the GEF-H1/RhoA interaction, and F1 as a fragment of a previously identified inhibitory domain within the C-terminus of GEF-H1 [17]. The latter strategy was used as the C-terminal GEF-H1 domain might be more specific as a naturally occurring inhibitor. However, neither of the two inhibitors affected the activity of p114RhoGEF, a highly homologous GEF for RhoA, or Dbl, after which the catalytically active GEF domain was named. While this does not rule out possible effects on other Rho GEFs, it does suggest that the inhibitors preferentially block GEF-H1. As F1 did not inhibit cell migration, a known function of GEF-H1 blocked by the intact C-terminal domain [17], fragmentation of the domain may have weakened the inhibitory activity. Hence, we only tested P5 in the in vivo disease model, which also reduced the risks of possible endotoxin contaminations in the inhibitor preparation as, unlike F1, did not have to be purified from bacterial extracts. Nevertheless, future studies will include more detailed experiments to determine inhibitor specificity and whether the two types of inhibitors show differential specificity.

We validated the GEF-H1 inhibitors in in vitro models of fibrosis and inflammation, as well as in a mouse model of retinal disease, experimental autoimmune uveitis. In this disease, TNFα is responsible for targeted and bystander tissue damage and can be suppressed by anti-TNFα therapies [57]. GEF-H1 is critical for TNFα responses in different experimental models [18,63]. Our data show that blocking GEF-H1 function inhibited disease progression. This correlated with inhibition of expression of glial fibrillary acidic protein (GFAP) expression, a marker of disease activity that indicates glial cell activation and inflammation. Blocking GEF-H1 function can thus effectively inhibit pathological responses relevant to multiple diseases affecting the retina as well as other organs. It will be important to test GEF-H1 inhibitors in the future and directly compare their effectiveness for the treatment of uveitis with current standard treatments such as dexamethasone, a treatment with considerable side effects [64,65]. Moreover, additional ocular disease models with likely involvement of GEF-H1 should be tested, such as other inflammatory models (e.g., conjunctivitis) or other common diseases affecting ocular endothelia and the RPE such as diabetic retinopathy and neovascular age-related macular degeneration (wet AMD).

GEF-H1 signaling has also been reported to be important for non-ocular diseases such as different types of cancers and metastasis. For example, a GEF-H1/PKD3 signaling pathway promotes the maintenance of triple-negative breast cancer stem cells [66]. GEF-H1 is also upregulated in colon cancer tissues and plays a key role in colon cancer metastasis through the GEF-H1-RhoA-MLC2 signaling pathway [67]. In hepatocellular carcinoma, GEF-H1 promotes cell motility via activation of RhoA signaling [68]. Thus, there are many possible therapeutic opportunities for GEF-H1 inhibitors for diseases affecting the eye and many other tissues. An important future target is to investigate GEF-H1 inhibitors as a therapeutic opportunity for different types of cancers and metastasis.

## 5. Patents

The work reported here has been filed for patent protection by UCL Business (UCLB) with the UK patent office (Patent No UK Intellectual Property Office; PCT/GB2020/050150).

## Data Availability

The primary data are available on request from the communicating authors.

## Supplementary Materials

The following supporting information can be downloaded at: www.mdpi.com/article/10.3390/cells11111733/s1, Figure S1. GEF-H1 inhibitors do not block p114RhoGEF-induced cell shape changes; Figure S2. TAT-F1 and TAT-P5 do not induce apoptosis or necrosis; Figure S3. GEF-H1 inhibitors and cell migration; Figure S4. GEF-H1 inhibitors attenuate cytoskeletal and junctional changes induced by thrombin; Figure S5. Clinical scoring of EAU experiments; Figure S6. GEF-H1 inhibitor TAT-P5 attenuates loss of blood/retinal barrier function; Figure S7. TAT-P5 does not affect the relative proportions of immune cell populations; Figure S8. Analysis of retinal histology and GFAP expression; Table S1. Primers used to generate CTD constructs; Table S2. Sequences of TAT-P5 inhibitor and negative control peptide.
