# Supplementary material for: Therapeutic Validation of GEF-H1 Using a De Novo Designed Inhibitor in Models of Retinal Disease
Source: Cells. 2022 May 24;11(11):1733. doi: 10.3390/cells11111733 (PMC9179336; doi:10.3390/cells11111733)
Supplement: Supplementary file 1 [file cells-11-01733-s001.zip › cells-1679023-supplementary.pdf]

Supplementary Figures and Tables – Mills et al.

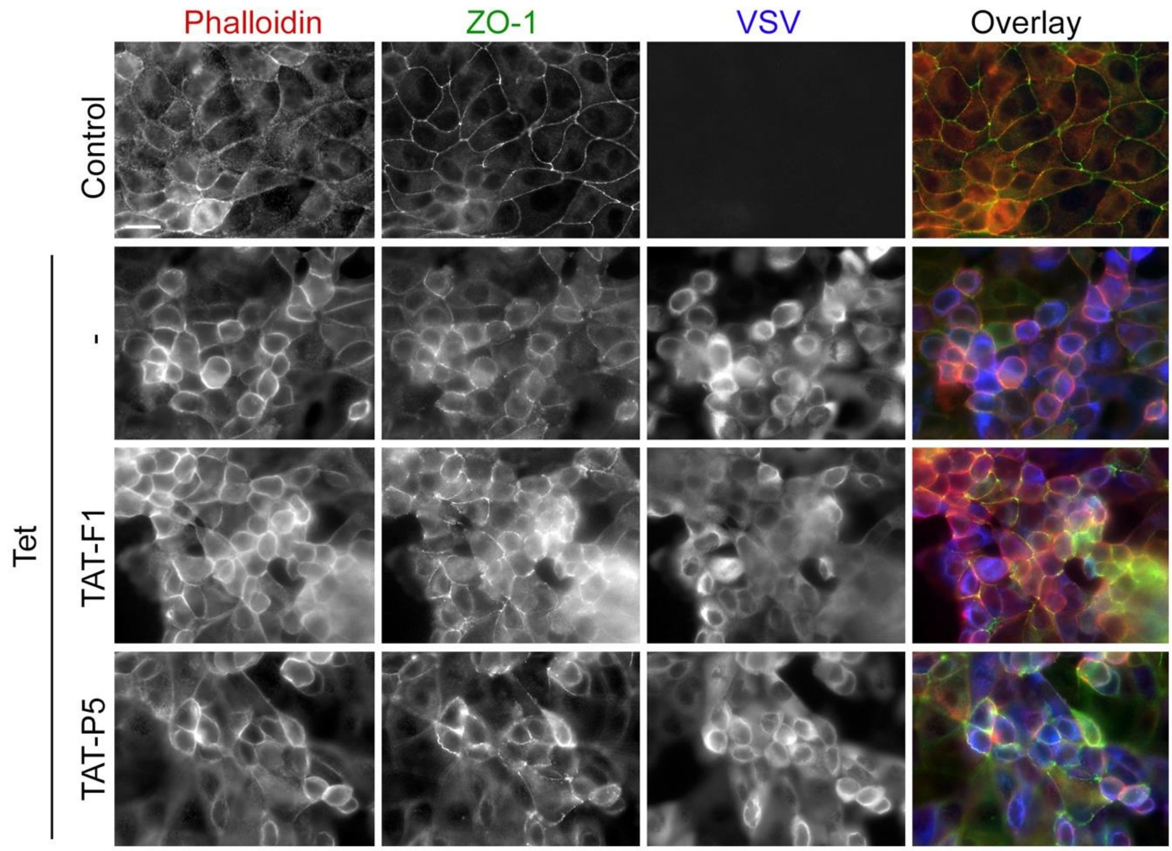

Figure S1. GEF-H1 inhibitors do not block p114RhoGEF-induced cell shape changes. MDCK cells conditionally expressing p114RhoGEF-VSV, a close homologue of GEF-H1 that induces cell contraction and cell rounding, were treated with tetracycline to induce expression of the GEF and were treated with TAT-F1 (4μM) or TAT-P5 (20μM). Cells were then fixed and stained for F-actin with fluorescent phalloidin and antibodies against ZO-1 and the VSV epitope. Note, contraction and apparent round of the cells is not affected by the GEF-H1 inhibitors. Scale bar, 20μm.

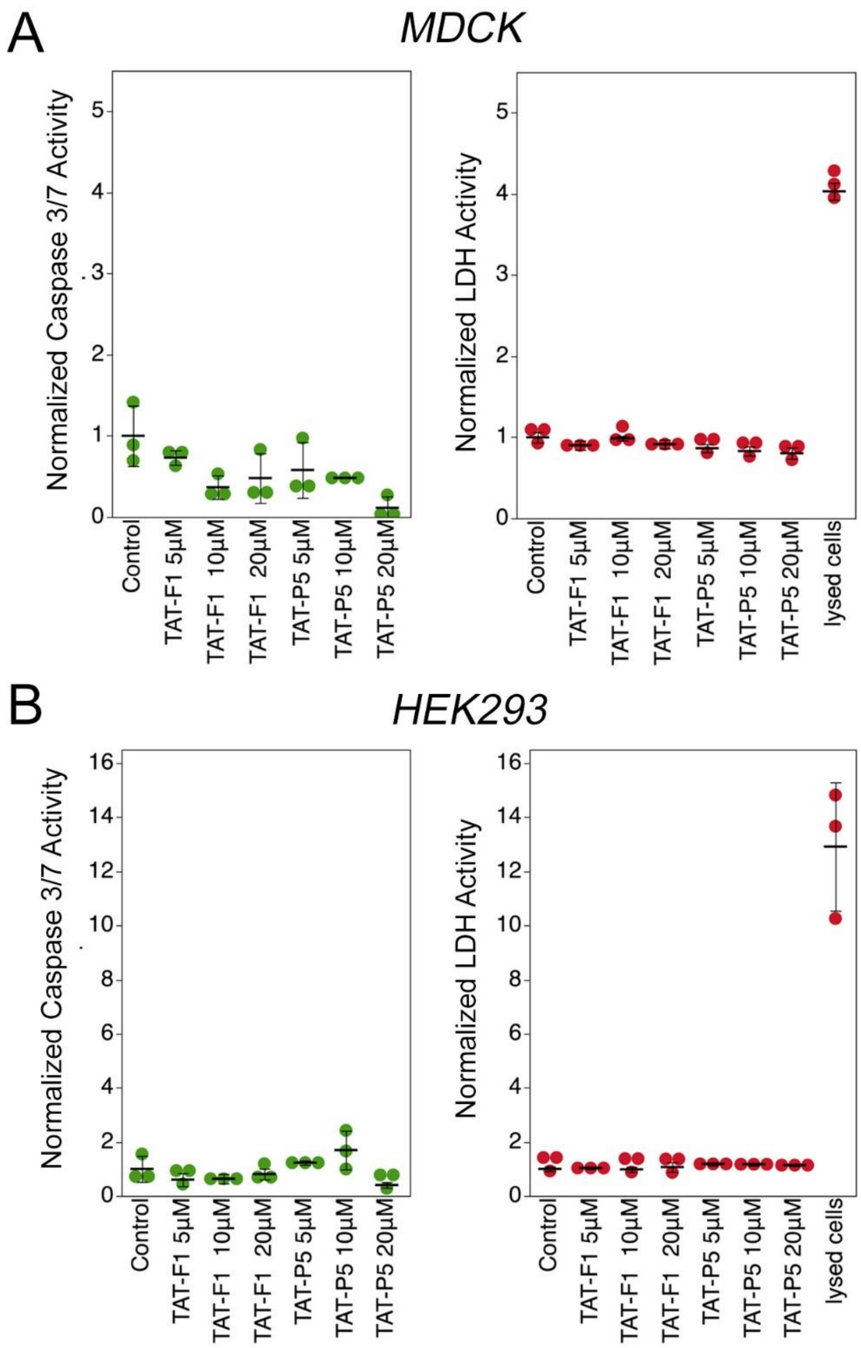

Figure S2. TAT-F1 and TAT-P5 do not induce apoptosis or necrosis.
MDCK **(A)** and HEK 293 **(B)** cells were incubated overnight with TAT-F1 or TAT-P5 at the concentrations indicated. Cell toxicity was then assessed by measuring caspase 3/7 activity in cell extracts and release of LDH into the medium. No significant cell toxicity was observed.

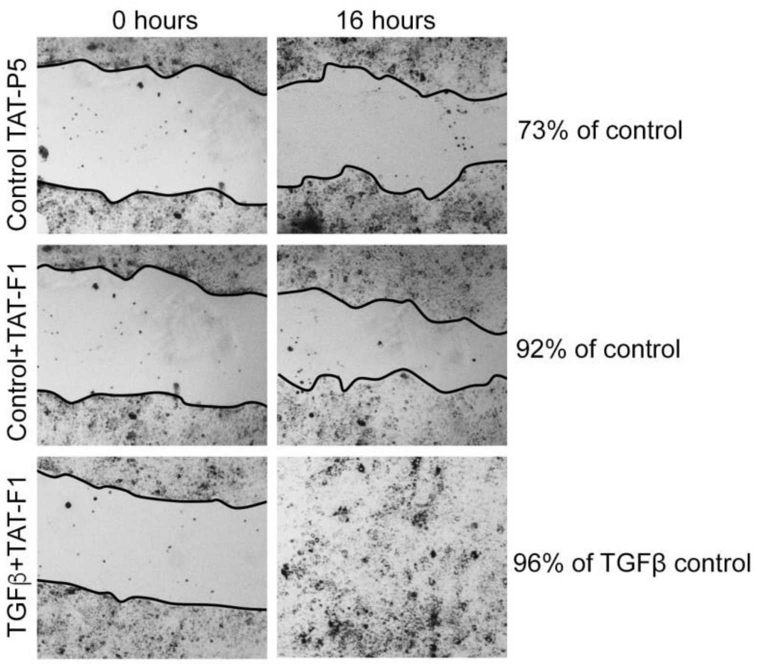

Figure S3. GEF-H1 inhibitors and RPE cell migration.
RPE cells were assayed for cell migration without or with TGFβ stimulation using a scratch wound assay in the presence of the indicated inhibitors. The images shown are derived from the same experiment as figure 5A, which also shows the relevant control images. The indicated percentage numbers are means derived from independent experiments and reflect of wound closure relative to the respective control.

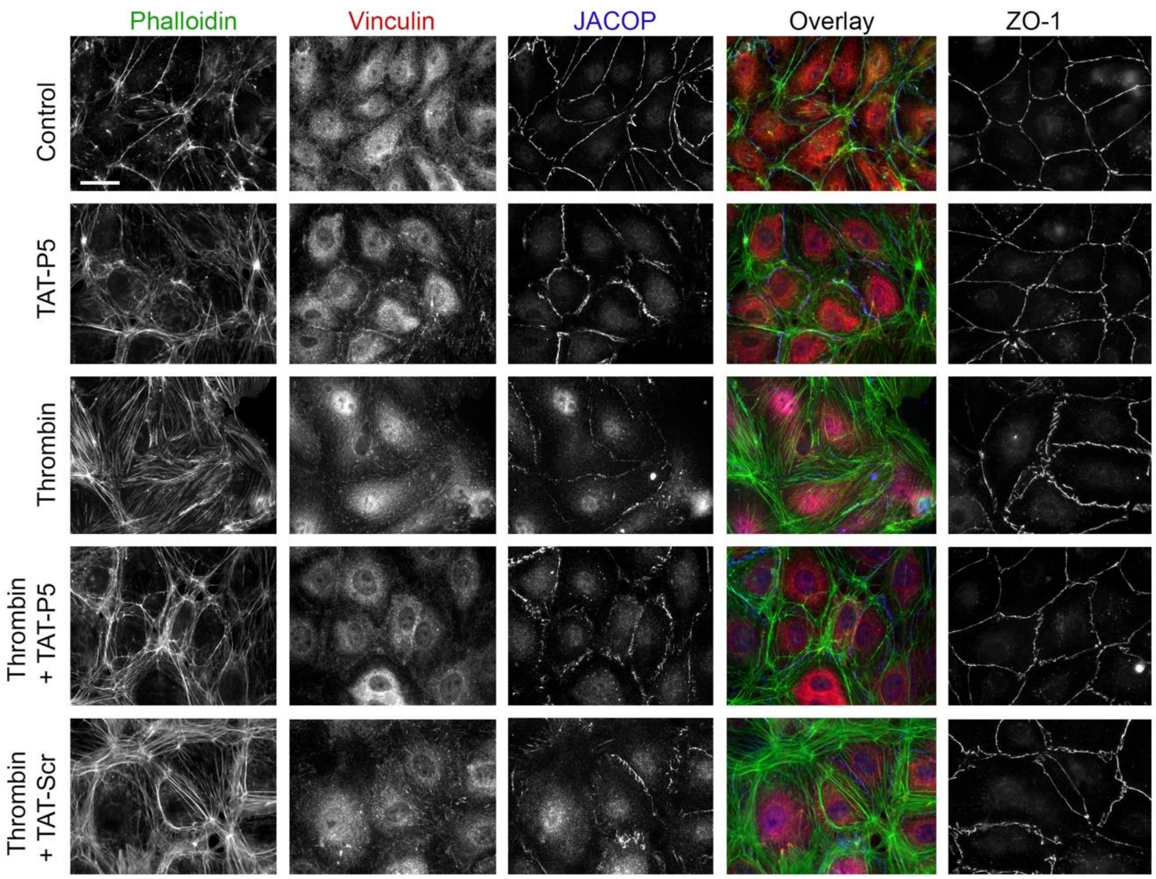

Figure S4. GEF-H1 inhibitors attenuate cytoskeletal and junctional changes induced by thrombin.

HDMEC primary endothelial cells were treated with Thrombin in the absence or presence of GEF-H1 inhibitor TAT-P5 or a control scrambled peptide. The cells were then fixed and stained as indicated. Scale bar, 20μm.

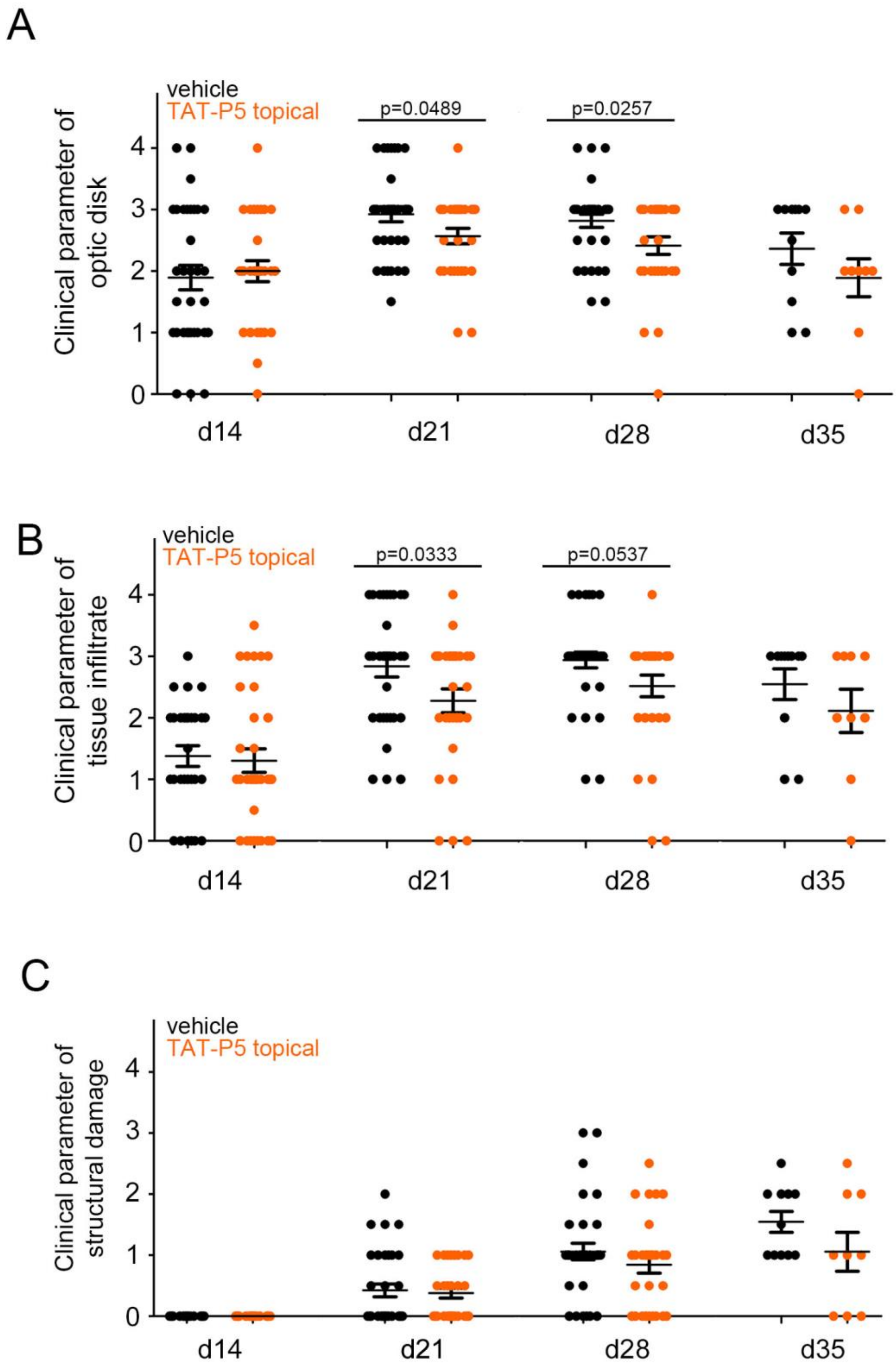

Figure S5. Clinical scoring of EAU experiments.
Shown are the individual clinical scores for optic disc neuropathy (A), retinitis (B) and structural damage (C) of the EAU experiments shown in figure 7 that were used to calculate the total clinical score (see figure 7E for the vasculitis scores). Time refers to days after immunization of the mice. ANOVA tests resulted in $p<0.0001$ for the optic disk (A) and tissue infiltrate (B) clinical score time course analyses.

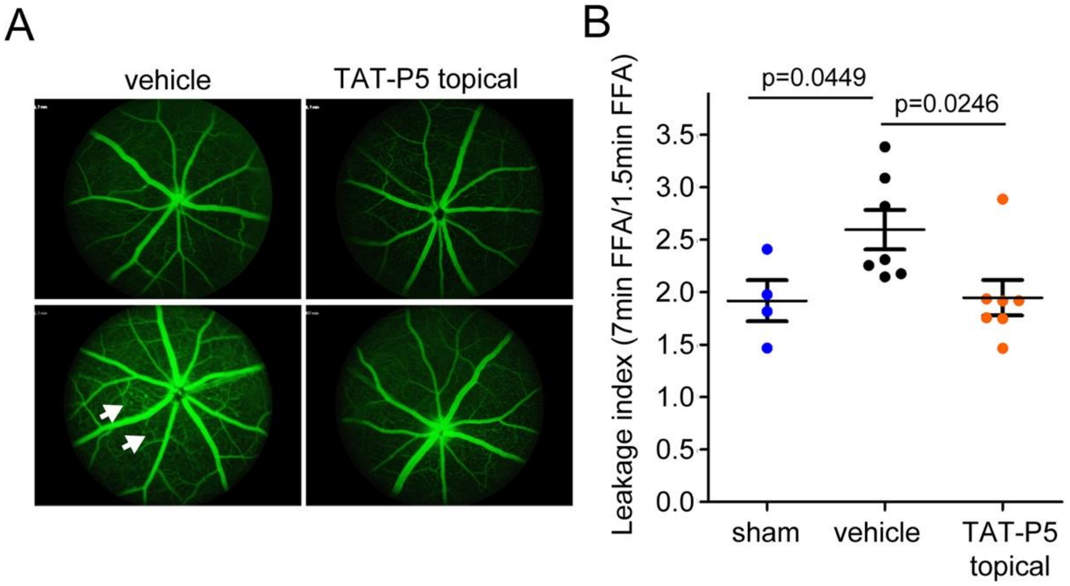

Figure S6. GEF-H1 inhibitor TAT-P5 attenuates loss of blood/retinal barrier function. Assessment of vascular leakage by fundus fluorescein angiography imaging at day 35 after immunization. Panel A shows images obtained 7 minutes after fluorescein injection. In the quantification in panel B, each data point represents one eye. Statistical significance was assessed using t-tests. Indicated are means ±SEM. Sham immunized mice served as negative controls. Arrows indicate points of vascular leakage.

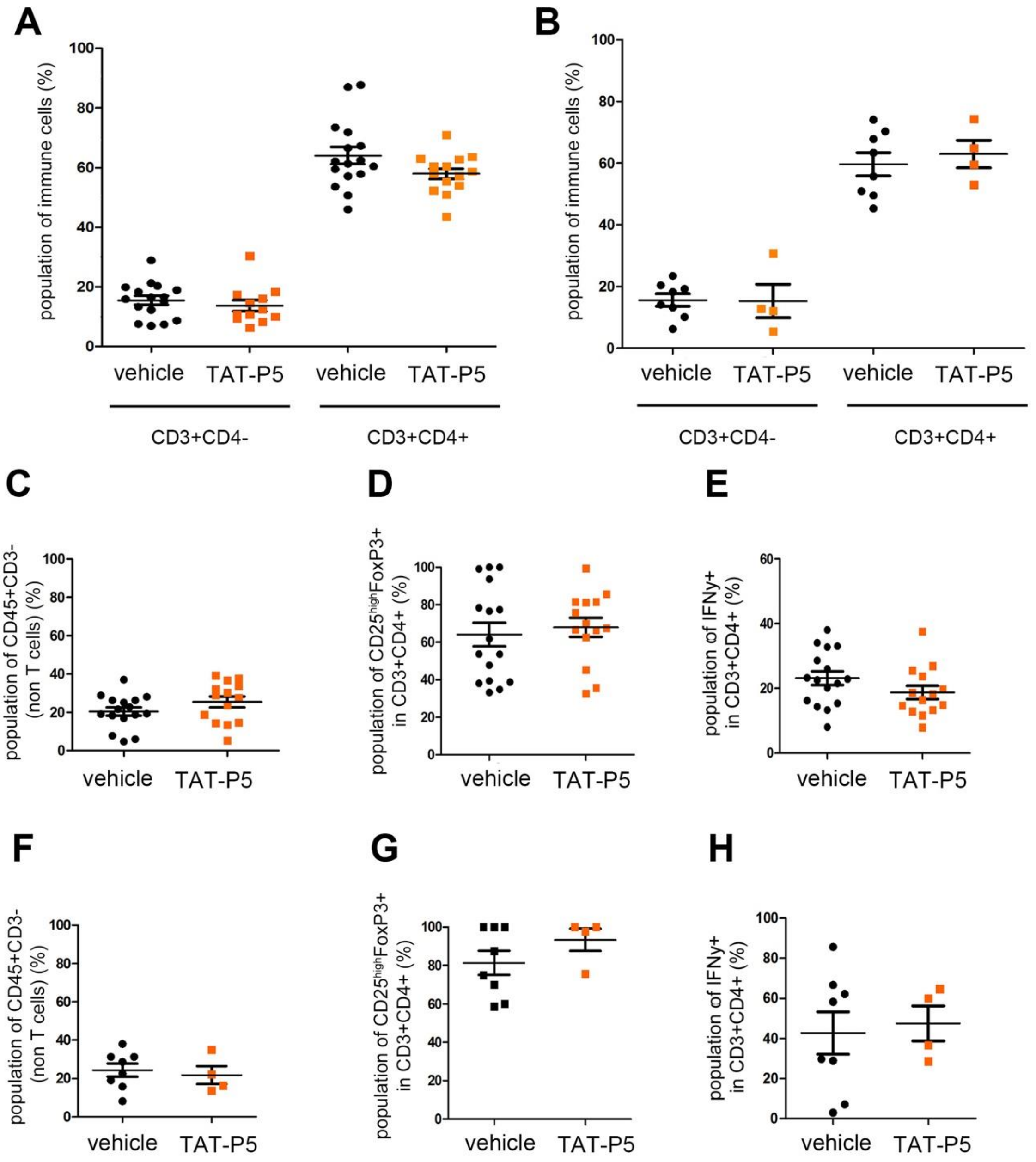

Figure S7. TAT-P5 does not affect the relative proportions of immune cell populations. EAU was induced, treated, and analysed as described in Figure 7 and 8. Single retinas were harvested at day 29 (A, C, D, E) and day 36 (B, F, G, H), respectively, and stained for immune cell markers. T cell populations ($CD3^{+}CD4^{-}$ and $CD3^{+}CD4^{-}$) were not different from each other (A, B) and no altered hematopoietic non-T cell response ($CD45^{+}$) was observed upon treatment (C, F). The regulatory T cells ($CD25^{high}FoxP3^{+}$, D + G) and T helper 1 cells ($IFN\gamma^{+}$, E + H) showed no difference between vehicle and treatment groups. Immune cell populations were investigated at day 29 or day 36. Data from at least three experiments were pooled and statistical significance was assessed using two-sided t-tests.

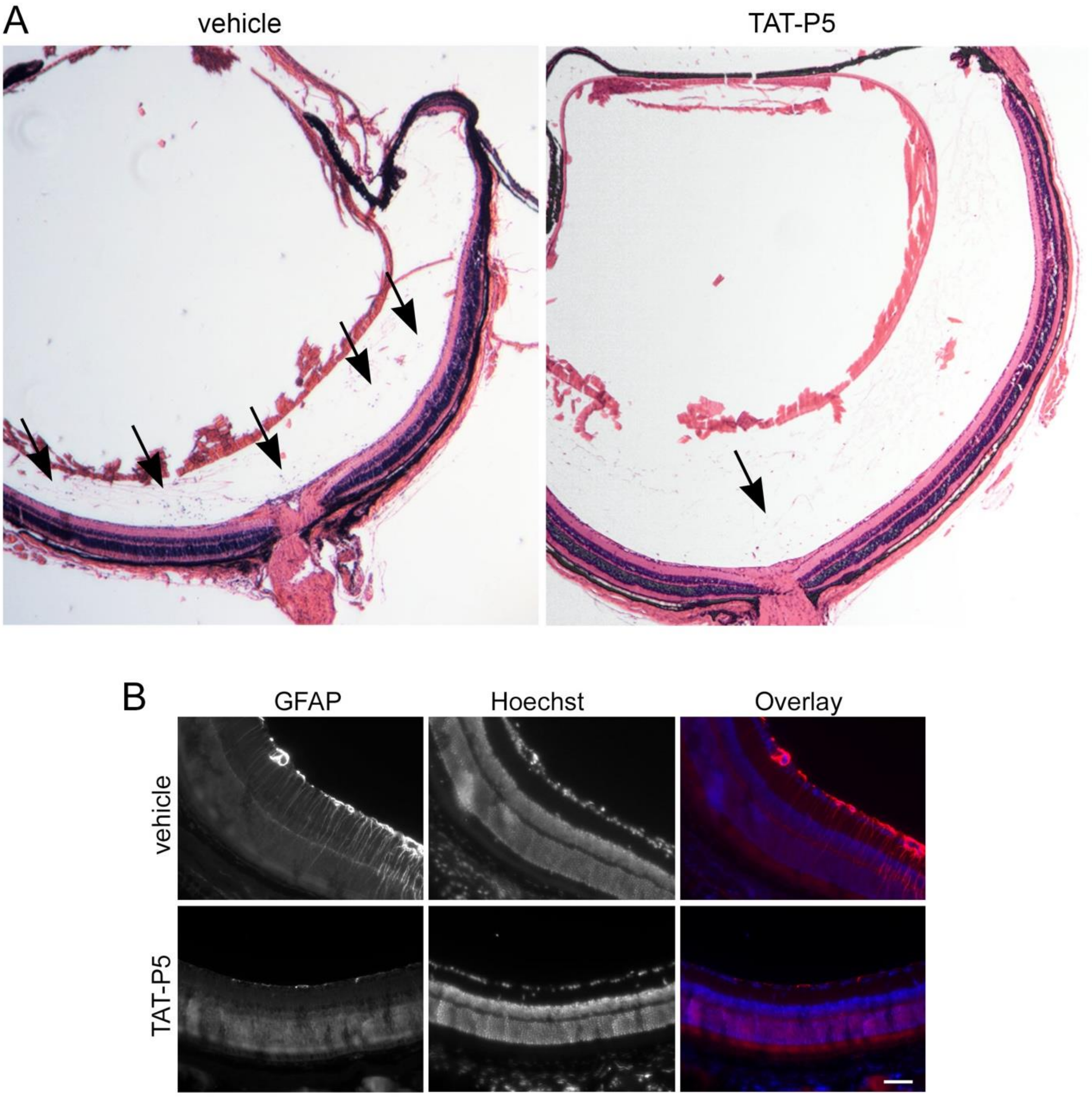

Figure S8. Analysis of retinal histology and GFAP expression.
(A) Shown are images of H&E-stained paraffin sections from vehicle control and TAT-P5 eyes that were analysed for the scoring shown in figure 8A. Arrows point to immune cells in the vitreous. (B) Retinal sections were stained for GFAP as in figure 8E. Shown is another set of images used for the quantification in figure 8F. Scale bars, 50μm.

Table S1. Primers used to generate CTD constructs

Construct	*Primers*
F1	F: AAAAACTTAAGATGAATTCCAGAGAGGACTTCCCCCTGATTG<br>R: AAAAAGTCGACCTAGCTAGCGTTTCTGGCCTCACCATTGGCTGTGA
F2	F: AAAAACTTAAGATGAATTCCAAAGACCTCCTGGTGGGGCCTG<br>R: AAAAAGTCGACCTAGCTAGCCAGCTCGGTGGCCTGCTTGTCAGG
F3	F: AAAAACTTAAGATGAATTCTCCAGGGATGGAGAGGCTGGCCGAGT<br>R: AAAAAGTCGACCTAGCTAGCTCGGCTGGGCTGTGGGGGCGTGAA
F4	F: AAAAACTTAAGATGAATTCAGCCTCCCTGCAGGCGATGCTCTGT<br>R: AAAAAGTCGACCTAGCTAGCGCTCTCTGAAACCATAGGCTCCC
TAT-A	F: GATCCATGGGCGGCTACGGCCGCAAGAAACGCCGCCAGCGCCGCCG<br>CGGTGGAGGCTCCGGCTATCCATATGACGTCCCAGACTATGCTGGCG<br>GCTCGAGG
TAT-B	R: AATTCCTCGAGCCGCCAGCATAGTCTGGGACGTCATATGGATAGCCG<br>GAGCCTCCACCGCGGCGGCGCTGGCGGCGTTTCTTGCGGCCGTAG<br>CCGCCCATG
TAT -F1	F: AAGCCGGCTCGAGCTCCAGAGAGGACTTCCCCCTGATTG<br>R: GCATGCATAGCGGCCGCCTAGTTTCTGGCCTCACCATTGGCTGTG

Table S2. Sequences of TAT-P5 inhibitor and negative control peptide

TAT-P5
GRKKRRQRRRPWQGQEDYDRLRPASYPDTDVI

TAT-Scr (scramble)
GRKKRRQRRRPWQDYIAEVPRSDYDLRPTGQD
